# Canal-to-Bone Ratio as an Independent Predictor of Hip Bone Mineral Density Scores: A Retrospective Analysis

**DOI:** 10.5152/eurasianjmed.2026.251164

**Published:** 2026-02-17

**Authors:** Salih Kaya

**Affiliations:** Erzurum City Hospital, Erzurum, Türkiye

**Keywords:** Bone mineral density, canal-to- bone ratio, hip fracture, osteoporosis

## Abstract

**Background:**

Hip fractures are a major consequence of osteoporosis. While dual-energy X-ray absorptiometry (DXA) is the gold standard for bone mineral density (BMD), radiographic indices may complement DXA. The canal-to-bone ratio (CBR)—the intramedullary canal diameter divided by the outer cortical diameter on plain radiographs—may reflect bone quality. We examined the relationship between CBR and BMD and whether CBR independently predicts hip T- and Z-scores.

**Methods:**

The study retrospectively analyzed 90 patients with femoral radiographs and DXA. Canal-to-bone ratio was measured 7 cm distal to the lesser trochanter. Lumbar and hip T-/Z-scores were recorded. Associations were tested with Pearson/Spearman correlations and linear regression; multivariable models adjusted for age.

**Results:**

Canal-to-bone ratio correlated negatively with hip BMD (T-score *r* = −0.63, *P* = .005; Z-score *r* = −0.65, *P* = .004), but not with lumbar scores (*P *> .20). Each 0.1 increment in CBR was associated with a 0.67-unit decrease in hip T-score and a 0.59-unit decrease in hip Z-score. After adjusting for age, CBR remained an independent predictor of hip T-score (β = −6.09, *P* = .012, *R*^2^ = 0.44) and hip Z-score (β = −5.59, *P* = .009, *R*^2^ = 0.43).

**Conclusion:**

Canal-to-bone ratio shows a strong, independent association with hip BMD. As a simple metric obtainable from routine radiographs, CBR may serve as a surrogate marker of hip bone quality and fracture risk, particularly in settings with limited DXA access.

**Main Points**

- The canal-to-bone ratio (CBR), measured on plain radiographs, showed a strong negative correlation with hip dual-energy X-ray absorptiometry T- and Z-scores.
- Canal-to-bone ratio was found to be an independent determinant of hip bone mineral density (BMD), even after age adjustment.
- No significant association was observed between CBR and lumbar BMD scores.
- Canal-to-bone ratio may serve as a simple and reproducible radiographic surrogate marker for hip bone quality and fracture risk assessment.

## Introduction

Osteoporotic hip fractures remain a major global health concern, particularly in elderly populations, where morbidity, mortality, and socioeconomic burden are substantial.^1^^-^^3^ Dual-energy X-ray absorptiometry (DXA) is the gold standard for evaluating bone mineral density (BMD), with results usually reported as T- and Z-scores.^4^^,^^5^ However, DXA availability and cost limit its widespread use in certain clinical contexts.^6^^,^^7^

Recently, morphometric indices derived from plain radiographs have gained attention as surrogate markers of bone quality.^8^^,^^9^ Among these, the canal-to-bone ratio (CBR)—defined as the ratio of intramedullary canal diameter to total femoral diameter—has emerged as a simple, reproducible parameter with potential diagnostic and prognostic value.^10^^-^^12^ While CBR has been associated with overall bone strength, its direct relationship with DXA-derived T- and Z-scores remains underexplored.

This study aimed to investigate the correlation between CBR and BMD scores (lumbar and hip T- and Z-scores) and to determine whether CBR serves as an independent predictor of hip BMD after adjustment for age.

## Material and Methods

### Study Design and Population

This retrospective cohort study included 90 patients (all with femoral radiographs and DXA results available) collected between 2021 and 2025 at a tertiary care institution. Patients with pathological fractures, prior hip surgery, or metabolic bone disease other than osteoporosis were excluded.

### Data Collection

**Figure 1.**
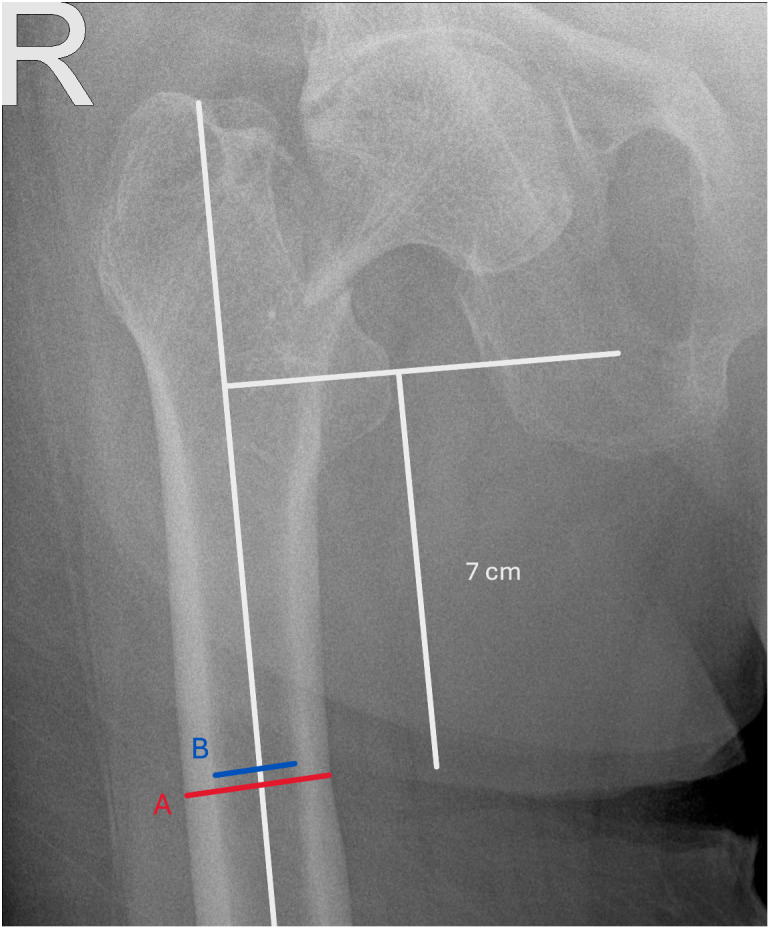
Measurement of the canal-to-bone ratio (CBR) on anteroposterior pelvic radiographs. The outer cortical diameter (red line, A) and the intramedullary canal diameter (blue line, B) were obtained at a standardized level located 7 cm distal to the lesser trochanter. The CBR was then calculated as the ratio of B to A.

For each patient, demographic and clinical data including age were recorded. Morphometric measurements were obtained from standardized anteroposterior pelvic radiographs. Canal-to-bone ratio was calculated at 7 cm distal to the lesser trochanter as the ratio of the intramedullary canal diameter (B) to the outer cortical (total femoral) diameter (A) (Figure 1).



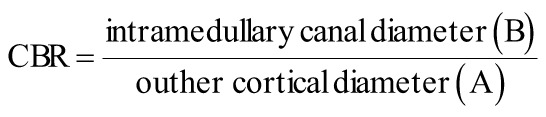



The measurement site, 7 cm below the lesser trochanter, was chosen to ensure consistency with prior morphometric studies that identified this level as representative of mid-diaphyseal cortical thickness while minimizing trochanteric overlap.^11^

Inter- and intraobserver reliability of CBR measurements showed excellent agreement (Intraclass Correlation Coefficient > 0.85).

BMD was assessed with DXA, and T- and Z-scores were obtained for the lumbar spine and hip. Hip BMD values were obtained from the total hip region, as provided by the densitometer software. All measurements were performed with a Stratos densitometer (DMS Imaging, France).

### Statistical Analysis

Descriptive statistics were presented as means ± standard deviations. The relationship between CBR and BMD scores was evaluated with Pearson and Spearman correlation tests, while linear regression was used to estimate the slope and the proportion of variance explained (*R*^2^). Subsequently, multivariate linear regression models were fitted to evaluate the independent effect of CBR on hip T- and Z-scores, adjusting for age. A two-tailed *P*-value <.05 was considered statistically significant.

All statistical analyses were conducted using Python (pandas, statsmodels, scipy) in Jupyter/Colab environment.

### Ethics Approval and Consent to Participate

The study adhered to the ethical standards of the Declaration of Helsinki. Ethical approval was granted by the Institutional Review Board of Health Science University prior to data collection (Approval No: 2025/08-223, Date: 10/07/2025), and verbal informed consent was obtained from all participants.

## Results

The cohort included 90 patients (mean age: ~74 years). All had DXA-derived lumbar and hip BMD values and corresponding morphometric measurements (Table 1).

Correlation analyses showed that CBR showed a significant correlation with hip BMD scores. For hip T-scores, the correlation coefficients were Pearson *r* = −0.63 (*P* = .0048) and Spearman ρ = −0.68 (*p* = 0.0018), while for hip Z-scores, the coefficients were Pearson *r* = −0.65 (*P* = .0038) and Spearman ρ = −0.62 (*P* = .0059). These findings indicate moderate-to-strong, negative, and statistically significant correlations. In contrast, lumbar T- and Z-scores demonstrated only weak, non-significant associations with CBR (*P* > .20) (Table 2).

In univariate regression, a 0.1 increase in CBR was associated with a 0.67 decrease in hip T-score and a 0.59 decrease in hip Z-score (*P* < .01). After adjustment for age, CBR remained a significant independent predictor, with β = −6.09 (*P* = .012, *R*^2^ = 0.44) for hip T-score and β = −5.59 (*P *= .009, *R*^2^ = 0.43) for hip Z-score. Multivariate regression results are summarized in Table 3.

## Discussion

The present study demonstrates that CBR is strongly and independently associated with hip T- and Z-scores, whereas lumbar scores do not show significant correlations. Age did not significantly affect these associations, indicating that CBR reflects bone quality independently of chronological age. These findings suggest that CBR may serve as a reliable surrogate marker of hip bone quality, reflecting cortical thinning and canal expansion processes characteristic of osteoporosis. This inverse correlation can be physiologically explained by endosteal resorption and canal widening that accompany cortical thinning in osteoporotic bone, resulting in reduced cortical support and lower BMD.

Previous studies have highlighted the utility of femoral morphometric indices, such as canal-to-calcar ratio or Singh index, but their reproducibility and predictive accuracy remain debated.^13^^-^^15^ Our findings support CBR as a simpler and more quantitative alternative, with direct statistical association to hip BMD. Moreover, our *R*^2^ values (~0.43-0.44) are comparable to those reported for other radiographic indices such as the canal-calcar ratio or cortical thickness index (typically 0.30-0.50 in similar cohorts), suggesting adequate explanatory power.^10^^-^^12^^,^^15^

The lack of correlation with lumbar scores may be due to degenerative changes and vertebral structural variations that limit lumbar DXA accuracy, a commonly cited limitation in previous studies.^16^^-^^18^

Clinically, these results emphasize the potential role of plain radiographs in osteoporosis screening, particularly in resource-limited settings where DXA is not readily available. Canal-to-bone ratio could be integrated into radiology reports to provide additional information on bone fragility.

The present study has certain limitations. Although the sample size is reasonable for a single-center retrospective analysis, it may still limit the generalizability of the findings. The retrospective design may also have introduced selection bias. Furthermore, potential confounding factors such as sex, body mass index, and comorbidities were not adjusted for, which could have influenced the outcomes. Finally, because this analysis was cross-sectional, causal relationships could not be determined. To confirm these findings and define standardized CBR thresholds for predicting osteoporosis and fracture risk, large-scale, prospective, multicenter studies in more diverse populations are needed.

The CBR, easily measurable from plain radiographs, is a significant independent predictor of hip T- and Z-scores. Unlike lumbar values, hip BMD demonstrates robust associations with CBR, highlighting its clinical value in osteoporosis assessment and fracture risk stratification.

## Figures and Tables

**Table 1.** Demographic and Clinical Characteristics of the Study Cohort

Variable	Value
Number of patients	90
Mean age (years)	74.8 ± 6.3
Sex (female/male)	68/22
Lumbar T-score	−2.48 ± 1.17
Hip T-score	−2.07 ± 0.81
Lumbar Z-score	−0.32 ± 1.22
Hip Z-score	−0.59 ± 0.7
CBR	0.56 ± 0.077

CBR = Canal-to-Bone Ratio; Values are presented as mean ± standard deviation.

**Table 2.** Correlation of CBR with BMD Scores

Outcome	N	Pearson *r*	*P*	Spearman rho	*P*
Lumbar T-score	90	−0.314	.204	−0.259	.299
Hip T-score	90	−0.634	.004*	−0.681	.002*
Lumbar Z-score	90	−0.305	.218	−0.232	.355
Hip Z-score	90	−0.645	.003*	−0.621	.006*

CBR = Canal-to-Bone Ratio; BMD = Bone Mineral Density. * *P* < .05 indicates statistical significance.

**Table 3.** Multivariate Regression Analysis of CBR and Hip BMD Scores

Outcome	Predictor	β coefficient	*P*-value	*R* ^2^
Hip T-score	CBR	−6.09	.012*	0.44
Hip T-score	Age	−0.025	.348	
Hip Z-score	CBR	−5.59	.009*	0.43
Hip Z-score	Age	−0.011	.632	

CBR = Canal-to-Bone Ratio; BMD = Bone Mineral Density. Regression coefficients (β) represent the effect per unit increase in predictor. * *P* < .05 indicates statistical significance.

## Data Availability

The data that support the findings of this study are available on request from the corresponding author.
